# The Active Subunit of the Cytolethal Distending Toxin, CdtB, Derived From Both *Haemophilus ducreyi* and *Campylobacter jejuni* Exhibits Potent Phosphatidylinositol-3,4,5-Triphosphate Phosphatase Activity

**DOI:** 10.3389/fcimb.2021.664221

**Published:** 2021-03-29

**Authors:** Grace Huang, Kathleen Boesze-Battaglia, Lisa P. Walker, Ali Zekavat, Zachary P. Schaefer, Steven R. Blanke, Bruce J. Shenker

**Affiliations:** ^1^Department of Basic and Translational Sciences, University of Pennsylvania School of Dental Medicine, Philadelphia, PA, United States; ^2^Department of Microbiology, University of Illinois, Urbana, IL, United States; ^3^Pathobiology Department, University of Illinois, Urbana, IL, United States; ^4^Biomedical and Translational Sciences Department, University of Illinois, Urbana, IL, United States

**Keywords:** cytolethal distending toxin, host-parasite interactions, lymphocytes, toxins, pathogenesis, cell cycle arrest, apoptosis

## Abstract

Human lymphocytes exposed to *Aggregatibacter actinomycetemcomitans* (Aa) cytolethal distending toxin (Cdt) undergo cell cycle arrest and apoptosis. In previous studies, we demonstrated that the active Cdt subunit, CdtB, is a potent phosphatidylinositol (PI) 3,4,5-triphosphate phosphatase. Moreover, AaCdt-treated cells exhibit evidence of PI-3-kinase (PI-3K) signaling blockade characterized by reduced levels of PIP3, pAkt, and pGSK3β. We have also demonstrated that PI-3K blockade is a requisite of AaCdt-induced toxicity in lymphocytes. In this study, we extended our observations to include assessment of Cdts from *Haemophilus ducreyi* (HdCdt) and *Campylobacter jejuni* (CjCdt). We now report that the CdtB subunit from HdCdt and CjCdt, similar to that of AaCdt, exhibit potent PIP3 phosphatase activity and that Jurkat cells treated with these Cdts exhibit PI-3K signaling blockade: reduced levels of pAkt and pGSK3β. Since non-phosphorylated GSK3β is the active form of this kinase, we compared Cdts for dependence on GSK3β activity. Two GSK3β inhibitors were employed, LY2090314 and CHIR99021; both inhibitors blocked the ability of Cdts to induce cell cycle arrest. We have previously demonstrated that AaCdt induces increases in the CDK inhibitor, p21^CIP1/WAF1^, and, further, that this was a requisite for toxin-induced cell death *via* apoptosis. We now demonstrate that HdCdt and CjCdt also share this requirement. It is also noteworthy that p21^CIP1/WAF1^ was not involved in the ability of the three Cdts to induce cell cycle arrest. Finally, we demonstrate that, like AaCdt, HdCdt is dependent upon the host cell protein, cellugyrin, for its toxicity (and presumably internalization of CdtB); CjCdt was not dependent upon this protein. The implications of these findings as they relate to Cdt’s molecular mode of action are discussed.

## Introduction

Cytolethal distending toxins (Cdt) represent a conserved and highly distributed family of toxins produced by more than 30 γ- and ϵ- Proteobacteria. Collectively, these bacteria contribute to the pathogenesis of a range of chronic infections and inflammatory disorders that affect mucocutaneous tissue (reviewed in Smith and Bayles, 2006; Scuron et al., 2016). Although Cdts are produced by a diverse group of pathogens, there is considerable similarity amongst the individual Cdts with respect to their range of toxic activities; these include induction of both cell cycle arrest and apoptosis in proliferating cell populations and induction of pro-inflammatory responses in macrophages (Smith and Bayles, 2006; Guerra et al., 2011; Scuron et al., 2016). Consistent with these observations is the finding that the active Cdt subunit, CdtB, exhibits considerable amino acid sequence conservation within the family of Cdts (Pons et al., 2019). Based upon these observations it is reasonable to expect that the individual CdtB subunits that comprise the diverse group of Cdt holotoxins would also share a similar mechanism of action.

Two molecular modes of action have been proposed to account for CdtB’s ability to induce both cell cycle arrest and apoptosis. The long-standing paradigm is that CdtB, functioning as a DNase, induces DNA damage that initially triggers activation of the G2/M checkpoint and, in turn, induces cell cycle arrest and eventually apoptosis (Cortes-Bratti et al., 2001a; Frisan et al., 2002; Thelestam and Frisan, 2004). In some instances, it has been proposed that cells surviving exposure to Cdt are susceptible to a secondary effect leading to genetic instability (Martin and Frisan, 2020). More recently, we demonstrated a new paradigm for the molecular mode of action for the *Aggregatibacter actinomycetemcomitans* Cdt (AaCdt) in which CdtB functions as a potent lipid phosphatase, depletes cells of the signaling lipid, phosphatidylinositol-3,4,5-triphosphate (PIP3), and thereby induces blockade of the ubiquitous PI-3K signaling pathway known to govern both cell proliferation and survival as well as a number of other cell functions (Shenker et al., 2007; Shenker et al., 2011; Shenker et al., 2014; Scuron et al., 2016; Shenker et al., 2016). The evidence for and against these modes of action has been reviewed in several recent articles (Guerra et al., 2011; Scuron et al., 2016; Pons et al., 2019). It is worth noting that both DNase and PIP3 phosphatase function as phosphoesterases and belong to a larger family of metalloenzymes each of which functions as a phosphoesterase (Dlakic, 2000; Dlakic, 2001); the specific function of each of these enzymes is likely to be largely dependent upon accommodation of substrates within their active site. Thus, it is not surprising that CdtB shares structural homology with both DNase I and inositol polyphosphate-5-phosphatase (IP5P), all members of the phosphoesterase superfamily (Shenker et al., 2007).

As noted above, we have reported extensively on the ability of CdtB derived from AaCdt to function as a PIP3 phosphatase and induce PI-3K signaling blockade. Importantly, we have demonstrated the requirement for this enzymatic activity and concomitant PI-3K signaling blockade in mediating both G2 arrest and apoptosis in human lymphocytes (Shenker et al., 2007; Shenker et al., 2016a; Shenker et al., 2016b). Moreover, we have demonstrated that as a consequence of Cdt-induced PI-3K blockade, the downstream kinase, glycogen synthase kinase 3β (GSK3β), is activated; inhibitors of this kinase block toxin-induced cell cycle arrest (Shenker et al., 2016). Another significant finding was that Cdt-induced increases in the intracellular levels of the cyclin-dependent kinase (CDK) inhibitor known as CDK-interacting protein (Cip1) and wild-type p53-activated fragment 1 (WAF1) (p21^CIP1/WAF1^) (Shenker et al., 2020); repressed expression of this regulatory protein blocks lymphocyte susceptibility to toxin-induced apoptosis.

The goal of this study was to determine if PIP3 phosphatase activity was also expressed by and critical to lymphocyte toxicity of other Cdts. In this study, we have assessed *Hemophilus ducreyi* Cdt (HdCdt) and *Campylobacter jejuni* Cdt (CjCdt) for their ability to both exhibit lipid phosphatase activity and induce PI-3K signaling blockade in lymphocytes. Furthermore, HdCdt and CjCdt were evaluated for similarities to AaCdt with respect to their reliance on key proteins critical to toxicity; these include GSK3β, p21^CIP1/WAF1^, and cellugyrin. It should be noted that HdCdt was selected because its CdtB subunit shares >90% homology with AaCdtB. In contrast, the CdtB subunit from CjCdt shares only limited amino acid sequence and structural homology with AaCdt and HdCdt; it shares greater homology with CdtB derived from *Escherichia coli* Cdt.

## Methods and Materials

### Expression and Purification of Recombinant AaCdt, HdCdt, and EcCdt

*In vitro* expression of AaCdt peptides was performed using the Rapid Translation System (RTS 500 ProteoMaster, Sapphire North America; Ann Arbor, MI) as previously described (Shenker et al., 2005). Reactions were run according to the manufacturer’s specification using 10-15 μg of template DNA. After 20 hrs at 30°C, the reaction mix was removed and the expressed Cdt peptides were purified by metal ion affinity chromatography (Shenker et al., 2005). Construction and expression of the plasmid containing the *cdt* genes for the Cdt holotoxin (pUCAacdtABChis) have previously been reported (Boesze-Battaglia et al., 2009). The histidine-tagged holotoxin was isolated by ion affinity chromatography.

HdCdt and CjCdt peptides were expressed using plasmid constructed in the SRB lab and purified as previously described (Eshraghi et al., 2010). Briefly, *E. coli* BL21 were transformed with Cdt-expressing plasmid in Luria-Bertani broth and expression induced (OD_600nm_: 0.4-0.6) with isopropyl 1-thio-β-D-galactopyranoside (Millipore Sigma Chemical Co; St. Louis, MI). Three hr later cells were harvested, resuspended in PBS containing 8 M urea and disrupted by sonication. Following clarification by centrifugation, Cdt subunits were purified using metal ion affinity chromatography [Chelating Sepharose Fast Flow; ThermoFisher; Waltham, MA)] and dialyzed as previously described (Eshraghi et al., 2010). The purity of all peptides were evaluated by SDS-PAGE and found to be >95% pure.

### Cell Culture, Assessment of Cell Cycle Arrest and Apoptosis

The T-cell leukemia cell line Jurkat (E6-1) was maintained as previously described in RPMI 1640 supplemented with 10% FBS, 2 mM glutamine, 10 mM HEPES, 100 U/ml penicillin and 100 μg/ml streptomycin (Shenker et al., 2004). Cells were harvested in mid-log growth phase and plated as described below. Jurkat^p21CIP1/WAF1-^ and Jurkat^Cg-^ cells were prepared using CRISPR/CAS9 technology; the preparation and characterization of these cells has been previously described (Boesze-Battaglia et al., 2017; Shenker et al., 2020). Both cell lines were maintained in medium containing puromycin (1 μg/ml); experiments were conducted in medium without puromycin.

To measure Cdt-induced cell cycle arrest, cells (1 x 10^6^) were incubated for 24 hr and then washed and fixed for 60 min with cold 80% ethanol (Shenker et al., 2005). The cells were stained with 10 µg/ml propidium iodide containing 1 mg/ml RNase (Millipore Sigma Co) for 30 min. Samples were analyzed on a Becton-Dickinson LSRII flow cytometer (BD Biosciences; San Jose, CA); a minimum of 15,000 events were collected for each sample; cell cycle analysis was performed using Modfit (Verity Software House; Topsham, ME).

Cdt-induced apoptosis was assessed following 48 hr incubation in the presence of medium or Cdt by assessing DNA fragmentation with the TUNEL assay (In Situ Cell Death Detection Kit; (Millipore Sigma Co) (Shenker et al., 2001). Cells were harvested and re-suspended in freshly prepared 4% formaldehyde and permeabilized with 0.1% Triton X-100 for 2 min at 4°C. Cells were then washed with PBS and incubated in a solution containing FITC labeled nucleotide and terminal deoxynucleotidyl transferase (TdT) according to the manufacturers specifications. FITC fluorescence was assessed by flow cytometry as previously described (Shenker et al., 2001).

### Western Blot Analysis of PI-3K Signaling Blockade and pH2AX

Cells were treated as described and solubilized in 20mM Tris-HCl buffer (pH7.5) containing 150 mM NaCl, 1mM EDTA, 1% NP-40, 1% sodium deoxycholate and protease inhibitor cocktail (Pierce). Samples (30 μg) were separated on 12% SDS-PAGE and then transferred to nitrocellulose. Membranes were blocked with BLOTTO and then incubated with one of the following primary antibodies for 18 hr at 4°C (Shenker et al., 1999): anti-pAkt (S473), anti pGSK3β (S9) (Cell Signaling Tech; Danvers, MA), anti-pH2AX (Abcam; Cambridge, MA) or anti-GAPDH (Santa Cruz Biotechnology). Membranes were incubated with goat anti-mouse immunoglobulin conjugated to horseradish peroxidase (Southern Biotech Technology). The Western blots were developed using chemiluminescence and analyzed by digital densitometry (Li-cor Odyssey; Li-Cor Bio; Lincoln, NE).

### Phosphatase Activity

Phosphatase activity was assessed by monitoring the dephosphorylation of PIP_3_ as previously described (Maehama et al., 2000; Shenker et al., 2007). Briefly, the reaction mixture (20 μl) consisted of 100 mM Tris-HCl (pH 8.0), 10 mM dithiothreitol, 0.5 mM diC16-phosphatidylserine (Avanti Polar Lipids; Alabaster, AL), 25 μM PIP_3_ (Echelon Inc; Salt Lake City, UT) and the indicated amount of CdtB. Appropriate amounts of lipid solutions were deposited in 1.5 ml tubes, organic solvent removed, the buffer added and a lipid suspension formed by sonication. Phosphatase assays were carried out at 37°C for 30 min; the reactions were terminated by the addition of 15 μl of 100 mM N-ethylmaleimide. Inorganic phosphate levels were then measured using a malachite green assay.

### Statistical Analysis

Mean ± standard error of the mean were calculated for replicate experiments. Significance was determined using a Student’s *t*-test. Differences between multiple treatments were compared by ANOVA paired with Tukey’s HSD posttest; a *P*-value of less than 0.05 was considered to be statistically significant.

## Results

**Figure 1:**
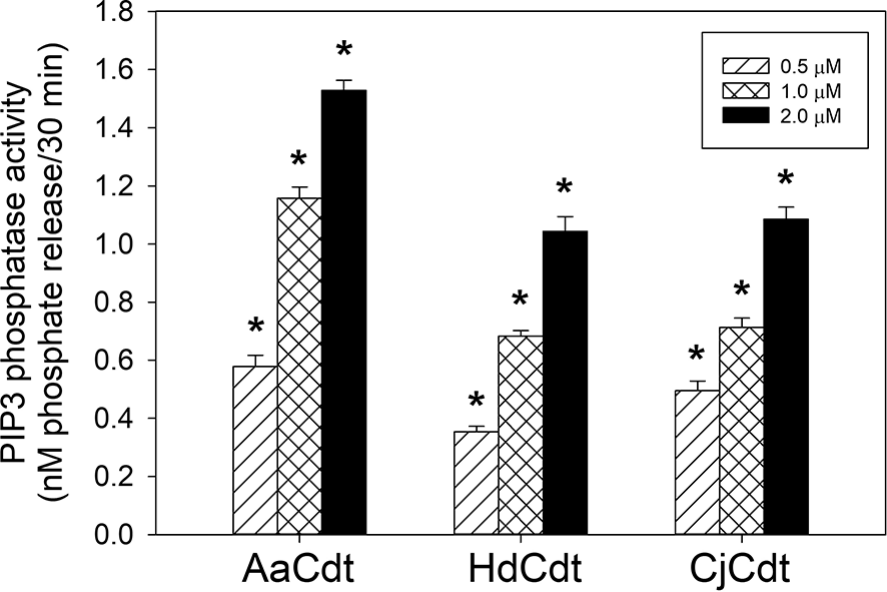
Assessment of the HdCdtB and CjCdtB subunits for PIP3 phosphatase activity. Varying amounts of CdtB derived from HdCdt and CjCdt were assessed and compared to AaCdtB for their ability to hydrolyze PIP3 as described in Materials and Methods. The amount of phosphate release was measured using a malachite green binding assay. Data are plotted as phosphate release (nM/30min;mean ± SEM) vs protein concentration. Results are derived from three experiments each performed in triplicate; *indicates statistical significance (p < 0.01) relative to background control samples (0.05 nM/30 min) not receiving protein samples.

First, we investigated whether CdtB-associated lipid phosphatase activity was restricted to AaCdtB or was instead a more universal property of the family of Cdts that is also exhibited by the active subunits of HdCdt and CjCdt. As shown in **Figure 1**, incubation of each CdtB subunit with the substrate PIP3 resulted in a dose-dependent (0.5-2.0 μM) release of phosphate. In the presence of 0.5, 1.0, and 2.0 μM HdCdtB, 0.35 ± 0.02, 0.68 ± 0.01, and 1.04 ± 0.05 nM phosphate was released, respectively; similar results were observed with CjCdtB: 0.49 ± 0.03 (0.5 μM), 0.71 ± 0.02 (1.0 μM), and 1.09 ± 0.04 nM (2.0 μM) phosphate release. These results indicate that both HdCdtB and CjCdtB exhibit PIP3 phosphatase activity that is comparable (~60-70%) to the enzymatic activity observed with AaCdtB: 58 ± 0.04, 1.16 ± 0.02, and 1.52 ± 003 nM phosphate release in the presence of 0.5,1.0, and 2.0 μM of the subunit.

**Figure 2:**
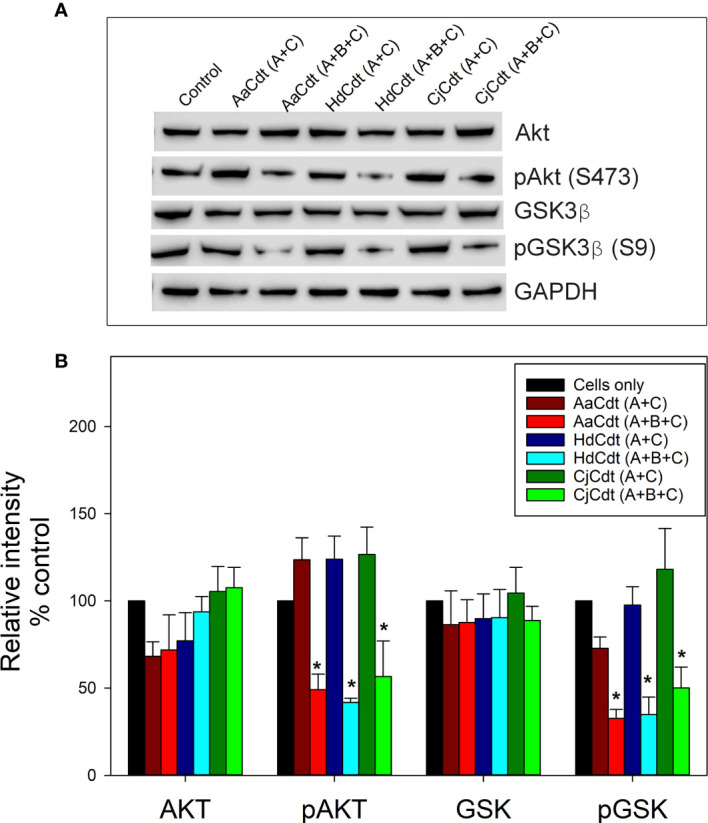
Comparison of the effect of HdCdt and CjCdt with AaCdt on phosphorylation of downstream components of the PI-3K signaling pathway. Jurkat cells were treated with AaCdt (AaCdtA/C: 0.5 nM; AaCdtB: 5.7 nM), HdCdt (HdCdtA/C: 0.2 nM; HdCdtB: 5.7 nM), and CjCdt (CjCdtA/C: 1.0 nM; CdtB: 5.7 nM) for 2 hr and then assessed for levels of Akt, pAkt(S473), GSK3ß and pGSK3ß(S9) by Western blot. **(A)** shows a representative Western blot; **(B)** shows the compiled results of four experiments (mean ± SEM); *indicates statistical significance p < 0.05 when compared to untreated control cells.

Following PIP3 depletion, control of cellular function by the PI-3K signaling pathway is achieved by altering the phosphorylation status of downstream kinases and phosphatases, resulting in either their activation or inactivation (Mitsiades et al., 2004; Huang and Sauer, 2010; Manning and Toker, 2017). Two of these key downstream regulatory components are the kinases protein kinase B (also known as Akt) and GSK3β; both of these kinases are phosphorylated when the PI-3K pathway is in an active state leading to Akt activation and GSK3β inactivation. Consistent with CdtB’s ability to function as a PIP3 phosphatase is its interference with PI-3K signaling which includes reduced phosphorylation of both Akt and GSK3β (Shenker et al., 2016). Specifically, we have previously demonstrated that lymphocytes exposed to AaCdt exhibit a significant reduction in not only the levels of PIP3 but also pAkt and pGSK3β at 2 and 4 hr. In the next series of experiments, we determined whether HdCdt and CjCdt also induced a similar blockade in the PI-3K pathway. Jurkat cells were treated with medium or the nontoxic binding subunits (CdtA + CdtC), serving as negative controls, and with the active complex (CdtA + CdtB + CdtC). A representative Western blot is shown in **Figure 2A** and demonstrates that cells exposed for 4 hr to medium alone or to only the binding subunits failed to exhibit significant changes in the levels of Akt, pAkt, GSK3β or pGSK3β. In contrast, when cells were treated with the active toxin complex (CdtA + CdtB + CdtC) for the same time period they exhibited reductions in both pAkt (S473) and pGSK3β (S9); total levels of Akt and GSK3β remained unaltered. **Figure 2B** represents the analysis of replicate experiments and indicates that in the presence of HdCdt, pAkt and pGSK3β levels were significantly reduced to 41.8 ± 2.4% and 56.6 ± 20.4% of control levels. Similarly, cells treated with CjCdt exhibited reduced levels of these kinases to 34.8 ± 10.0% (pAkt) and 50.1 ± 11.9% (pGSK3β) of control values. In previous studies, we have demonstrated, and confirm here, that Jurkat cells treated under identical conditions with AaCdt exhibited significantly reduced levels of both pAkt (45.5 ± 11.6%) and pGSK3β (47.6 ± 6.1%) (Shenker et al., 2016).

**Figure 3:**
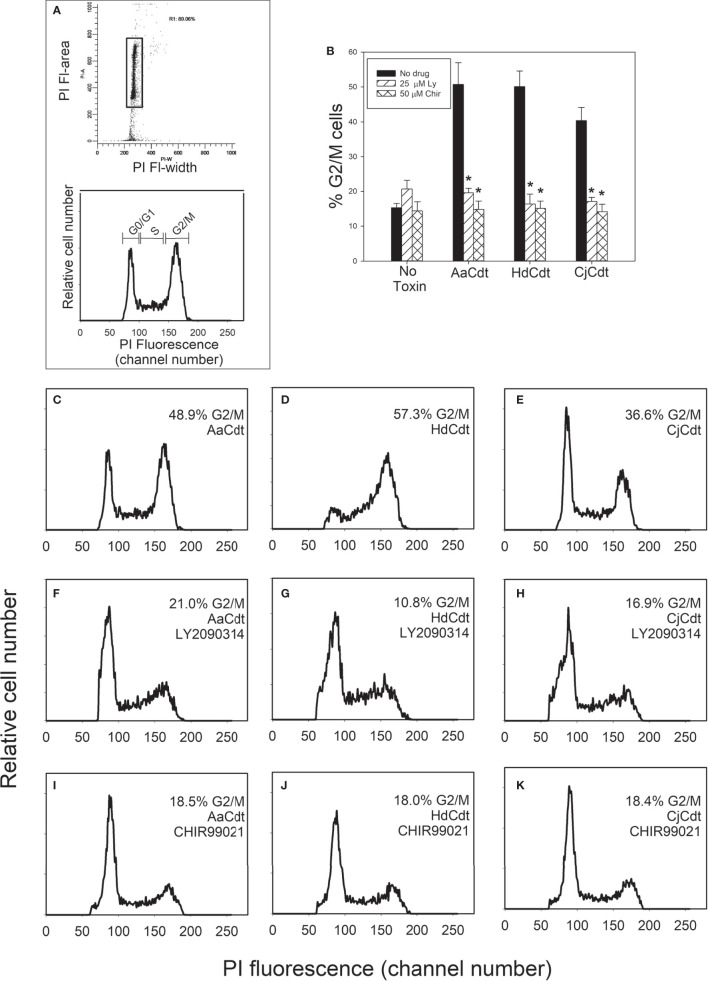
Assessment of GSK3β inhibitors for ability to block Cdt-induced cell cycle arrest. Jurkat cells were pre-treated with medium, 25 µM LY2090314, or 50 µM CHIR99021 for 1 hr followed by the addition of AaCdt (130 pM holotoxin), HdCdt (HdCdtA/C: 0.2 nM; HdCdtB: 0.3 nM) or CjCdt (CjCdtA/C: 1.0 nM; CjCdtB: 1.2 nM). Cells were harvested 24 hr later and analyzed for cell cycle distribution using propidium iodide and flow cytometry as described in Materials and Methods. **(A)** shows the gating strategy to identify cell cycle distribution. Debris and cell doublets were gated out using plots of propidium iodide fluorescence area versus width (top panel; rectangle); analytical gates were set to identify cells in the G0/G1, S and G2/M phases of the cell cycle based upon propidium iodide fluorescence (bottom panel). The percentage (mean ± SEM) of G2/M cells from three experiments, each performed in duplicate is shown in panel **(B)**; *indicates statistical significance (p<0.01) when compared to cells treated with toxin alone. Representative histograms are presented from a single experiment: panel **(C–E)** show the results from cells pre-treated with medium only, panel **(F–H)** show results from cells pre-treated with LY2090314, and panel **(I–K)** show results from cells pre-treated with CHIR99021. Cells exposed to medium alone exhibited 15.3 ± 2.1% G2/M cells. Panels **(C, F, I)** were treated with AaCdt, panels **(D, G, J)** were treated with HdCdt and panels **(E, J, K)** were treated with CjCdt.

Changes in the phosphorylation status of Akt and GSK3β differentially alter their kinase activities. Non-phosphorylated Akt is inactive and pAkt is active while phosphorylation of GSK3β has the opposite effect, converting the kinase to its inactive state. Indeed, we have demonstrated that Cdt-treated Jurkat cells exhibit a 200% increase in GSK3β kinase activity; furthermore, activation of GSK3β is a requisite for Cdt toxicity as inhibitors of this kinase block toxin-induced cell cycle arrest (Shenker et al., 2016). Therefore, we next determined if two GSK3β inhibitors, LY2090314 and CHIR99021, could also block HdCdt and CjCdt induced G2/M arrest (**Figures 3A–K**). Cells exposed to medium alone exhibited 15.3 ± 1.2% cells in the G2/M phases of the cell cycle; cells exposed to just the CdtA and CdtC subunits exhibited similar levels of cells in the G2/M phases (data not shown). As shown in Figs. 3B-3E, cells exposed to the active AaCdt, HdCdt, and CjCdt complexes (CdtA+CdtB+CdtC) demonstrated significant increases in cell cycle arrest with 50.7 ± 10.8%, 50.1 ± 7.7%, and 40.4 ± 6.5% cells in the G2/M phase, respectively. Pre-treatment of cells with the GSK3β inhibitor LY2090314 (panels 3B, 3F-3H) reduced the percentage of G2/M cells in the presence of Cdt to 19.6 ± 2.2% (AaCdt), 16.4 ± 4.9% (HdCdt), and 17.1 ± 0.2% (CjCdt). Similarly, pre-treatment with another GSK3β inhibitor, CHIR99021 (panels 3B, 3I-3K), also reduced the percentage of G2/M cells for all three toxins: 14.8 ± 4.1% (AaCdt), 15.1 ± 3.6% (HdCdt), and 14.2 ± 3.6% (CjCdt).

**Figure 4:**
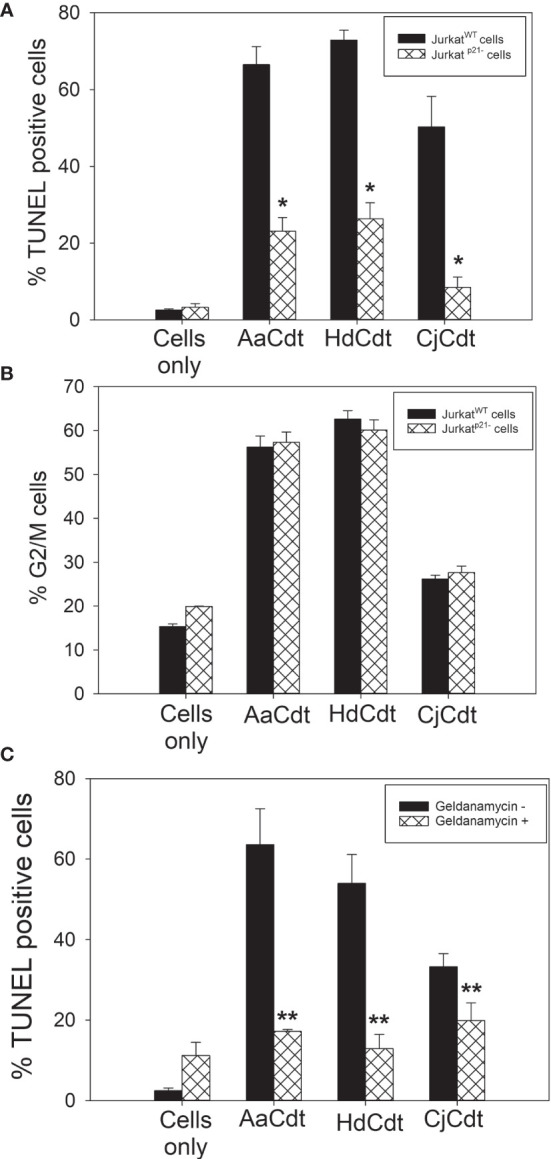
Assessment of the requirement for the CDK inhibitor p21^CIP1/WAF1^ in Cdt-induced toxicity. **(A)** Jurkat^WT^ cells and Jurkat^p21CIP1/WAF1-^ were treated with AaCdt, HdCdt and CjCdt (same concentrations as above) for 48hr and then analyzed for apoptotic cells by determining the percentage of TUNEL positive cells as detailed in Materials and Methods. The percentage of apoptotic cells is plotted for both Jurkat^WT^ (solid bars) and Jurkat^p21CIP1/WAF1-^ (cross hatched bars) cells and represent the mean ± SEM of three experiments. *indicates statistical significance (p < 0.01) when compared to Jurkat^WT^. **(B)** Jurkat^WT^ cells and Jurkat^p21CIP1/WAF1-^ were treated with AaCdt, HdCdt and CjCdt for 16hr and assessed for the percentage of cells in the G2/M phase of the cell cycle as described in Materials and Methods. The percentage of G2/M cells is plotted for both Jurkat^WT^ (solid bars) and Jurkat^p21CIP1/WAF1-^ (cross hatched bars) cells and represent the mean ± SEM of three experiments. **(C)** Jurkat cells were pre-treated with medium (solid bars) or Geldanamycin A [GA; (cross-hatched bars)] for one hr before the addition of Cdt. Cells were analyzed for apoptosis using the TUNEL assay 48 hr later; results represent the mean ± SEM of three experiments. **indicates statistical significance (p < 0.05).

Another key element in AaCdt-mediated toxicity involves the role of the CDK inhibitor p21^CIP1/WAF1^  (Shenker et al., 2020). Several studies, including our own, have demonstrated that within 4 hrs of exposure to Cdt, cells exhibit increased levels of p21^CIP1/WAF1^. Moreover, we have recently demonstrated that the increase in p21^CIP1/WAF1^ was a requisite step for Cdt-induced apoptosis (Shenker et al., 2020). Specifically, Jurkat cells deficient in p21^CIP1/WAF1^ expression (Jurkat^p21CIP1/WAF1-^) were found to be refractory to AaCdt-induced apoptosis. To determine if this dependence on p21^CIP1/WAF1^ extended to the other Cdts, we next compared the susceptibility of Jurkat wildtype cells (Jurkat^WT^) with Jurkat^p21CIP1/WAF1-^ cells to AaCdt, HdCdt and CjCdt-induced apoptosis. As shown in **Figure 4A**, Jurkat^WT^ cells treated with the active toxin complex of AaCdt,HdCdt and CjCdt exhibited a significant increase in the percentage of TUNEL positive (apoptotic) cells: 66.5 ± 4.7% (AaCdt) 72.9 ± 2.6% (HdCdt) and 50.2 ± 8.0% (CjCdt) over 2.6 ± 0.3% observed with control (untreated) cells. In contrast, Jurkat^p21CIP1/WAF1-^ cells exhibited fewer apoptotic cells when exposed to AaCdt (23.1 ± 3.6%), HdCdt (26.3 ± 4.1%) and CjCdt (8.4 ± 2.7%). Untreated cells exhibited 3.3 ± 1.0% apoptotic cells.

In our previous study, we did not report on the relationship between p21^CIP1/WAF1^ and Cdt-induced cell cycle arrest. In contrast to Cdt-induced apoptosis, we observed that p21^CIP1/WAF1^ expression was not required for Cdt-induced cell cycle arrest. As shown in **Figure 4B**, Jurkat^WT^ cells treated with Cdts exhibited 56.3 ± 2.5% (AaCdt), 62.6 ± 1.9% (HdCdt), and 26.1 ± 0.9% G2/M cells versus 15.3 ± 0.6% in untreated control cells. Jurkat^p21CIP1/WAF1-^ cells treated with the same dose of Cdts as the Jurkat^WT^ cells resulted in similar percentages of cells in the G2/M phases: 57.3 ± 2.3% (AaCdt), 60.1 ± 2.3% (HdCdt), and 27.6 ± 1.5% (CjCdt) compared to 19.9 ± 0.1% in untreated control cells.

Intracellular levels of p21^CIP1/WAF1^ are controlled transcriptionally, as well as post-translationally (Warfel and El-Deiry, 2013; Dutto et al., 2015). Previously, we demonstrated that p21^CIP1/WAF1^ mRNA levels were not significantly altered in AaCdt-treated lymphocytes (Shenker et al., 2020); instead, we observed that the increases were likely dependent upon post-translational modification as we have demonstrated a requisite role for the chaperone protein, HSP90 (Shenker et al., 2020). HSP90 has been shown to stabilize p21^CIP1/WAF1^ and prevent proteasomal degradation (Jascur et al., 2005; Diehl et al., 2009). Specifically, we have shown that geldanamycin A (GA), a HSP90 inhibitor, blocks both AaCdt-induced increases in p21^CIP1/WAF1^ and apoptosis. Based upon these observations, we utilized GA to determine if HSP90 was also involved in HdCdt- and CjCdt-induced apoptosis. As shown in **Figure 4C**, Jurkat^WT^ cells exposed to HdCdt and CjCdt exhibited 53.9 ± 7.2% and 33.2 ± 3.3% apoptotic (TUNEL positive) cells; control (untreated) cells exhibited 2.5 ± 0.4% TUNEL positive cells. Pre-treatment with GA reduced the percentage of apoptotic cells to 12.9 ± 3.5% (HdCdt) and 19.9 ± 4.4% (CjCdt). Similar to our previous results for AaCdt (Shenker et al., 2016) cells treated with AaCdt exhibited 63.5 ± 8.9% apoptotic; pretreatment with GA reduced the percentage to 11.2 ± 3.2% apoptotic cells.

**Figure 5:**
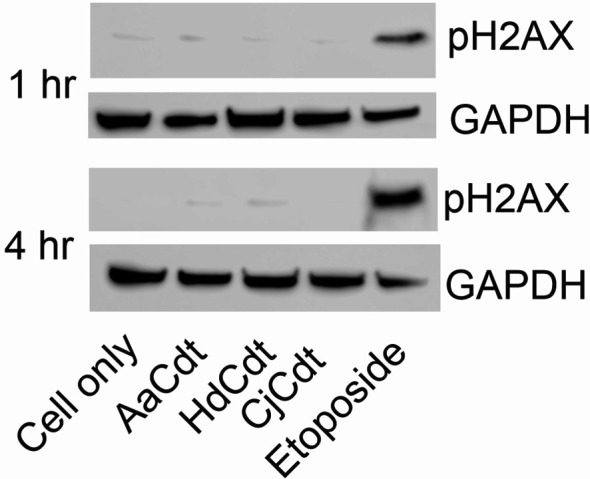
Comparison of the effects of AaCdt, HdCdt, and CjCdt on phosphorylation of H2AX. Jurkat^WT^ cells were incubated in the presence AaCdt, HdCdt, and CjCdt (same concentrations as above) for one and four hrs. Cells were then fractionated and analyzed by Western blot for the presence of pH2AX. Etoposide-treated cells were used as a positive control. A representative blot of three experiments is shown.

It should also be noted that upregulation of p21^CIP1/WAF1^ may also occur as a result of DNA damage (reviewed in Karimian et al., 2016) which ultimately leads to activation of the DNA damage response (DDR). DDR activation leads to concomitant activation of the G2/M checkpoint and cell cycle arrest. Indeed, several investigators have demonstrated that Cdts activate the DDR as exemplified by increased phosphorylation of the histone H2AX (pH2AX), a commonly employed measure of DDR activation. Increased pH2AX has been interpreted by several investigators as not only an indicator of DDR activation, but also as a surrogate for Cdt-mediated DNA damage (Bezine et al., 2014; Frisan, 2016). It should be noted, however, that we previously demonstrated that AaCdt-mediated p21^CIP1/WAF1^ upregulation in human lymphocytes is not linked to activation of the DDR; the doses of AaCdt required to induce cell cycle arrest (and apoptosis) do not induce increases of pH2AX (Shenker et al., 2006; Shenker et al., 2016; Shenker et al., 2020). Therefore, we also evaluated the effect HdCdt and CjCdt at the doses employed in this study for their ability to induce increases in pH2AX in lymphocytes. As shown in **Figure 5**, HdCdt and CjCdt, like AaCdt, failed to induce increased levels of pH2AX at these toxin concentrations at either 1 hr or 4 hr; exposure to etoposide was employed as a positive control.

**Figure 6:**
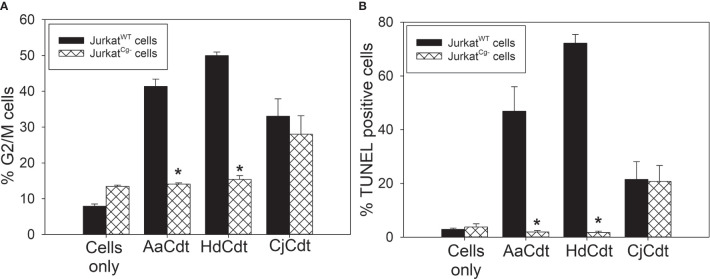
Comparison of HdCdt and CjCdt for the requirement of the host cell protein cellugyrin to elicit toxicity. **(A)** Jurkat^WT^ (solid bars) and Jurkat^Cg-^ (cross-hatched bars) cells were treated with AaCdt, HdCdt, and CjCdt as described above for 16 hr and then assessed for cell cycle distribution. Results show the percentage of cells in the G2/M phases of the cell cycle and are plotted as mean ± SEM of three experiments. **(B)** Jurkat^WT^ (solid bars) and Jurkat^Cg-^ (cross-hatched bars) cells were treated with AaCdt, HdCdt, and CjCdt for 48 hr and analyzed to determine the percentage of apoptotic cells using the TUNEL assay; results are plotted as the mean ± SEM of three experiments. *indicates statistical significance (p < 0.01) when compared to Jurkat^WT^ cells.

In the last series of experiments, we focused on recent observations made involving the earliest stages of AaCdt intoxication of host cells. Specifically, internalization of the CdtB subunit has been shown to be dependent upon the host cell protein, cellugyrin (synaptogyrin-2). Indeed, we have demonstrated that soon after exposure to Cdt, intracellular molecular complexes form that contain both CdtB and cellugyrin. Moreover, cells deficient in the expression of cellugyrin (Jurkat^Cg-^) were resistant to CdtB internalization and intoxication (Boesze-Battaglia et al., 2017; Boesze-Battaglia et al., 2020). Therefore, we explored whether this dependence on cellugyrin also extended to HdCdt and CjCdt. Jurkat^WT^ cells and Jurkat^Cg-^ cells were compared for their susceptibility to intoxication (both cell cycle arrest and apoptosis) by HdCdt and CjCdt. Cells were treated with toxin for 24 hr and then assessed for cell cycle distribution (**Figure 6A**). The percentages of Jurkat^WT^ cells in the G2/M phase of the cell cycle were 41.4 ± 2.0% (AaCdt), 50.0 ± 1.0% (HdCdt), and 33.0 ± 4.9% (CjCdt) compared with 7.9 ± 0.6% in untreated cells. In contrast, Jurkat^Cg-^ cells exhibited significant resistance to both AaCdt and HdCdt, exhibiting reduced cells in the G2/M phase: 14.1 ± 0.3% and 15.4 ± 1.0%, respectively; cells treated with CjCdt exhibited a consistent, but not statistically significant, reduction to 28.0 ± 5.1%. Control Jurkat^Cg-^ cells exhibited 13.5 ± 0.3% G2/M cells. Similar results were observed when cells were assessed for toxin-induced apoptosis (**Figure 6B**). Cells were treated with the same concentration of toxin as was employed for cell cycle analysis, but the analysis was performed at 48 hr. Jurkat^WT^ cells exhibited an increase in TUNEL positive cells when exposed to each of the Cdts: 46.9 ± 9.1% (AaCdt), 72.2 ± 3.3% (HdCdt), and 21.5 ± 6.6% (CjCdt) compared to 2.9 ± 0.5% in untreated control cells. In comparison, Jurkat^Cg-^ cells exposed to AaCdt and HdCdt exhibited a significant reduction in TUNEL positive cells: 1.9 ± 0.5% and 1.7 ± 0.5%, respectively; cells treated with CjCdt exhibited 20.8 ± 5.9%, similar to that observed with Jurkat^WT^ cells.

## Discussion

Cdts represent a family of exotoxins that fit the AB2 toxin model in which the binding (B) subunit is comprised of subunits CdtA and CdtC; the active (A) subunit is CdtB (Scuron et al., 2016). The focus of our ongoing investigation into Cdt has been to advance understanding of the virulence potential of AaCdt and ultimately define CdtB’s molecular mode of action. In this regard, many investigators have provided evidence in support of a role for CdtB-associated DNase activity in contributing to the underlying mode of action; the evidence (pro and con) for this mechanism has been reviewed in several recent publications (Cortes-Bratti et al., 2001; Frisan et al., 2002; Thelestam and Frisan, 2004; Scuron et al., 2016). In contrast, our recent studies have provided significant evidence supporting a new paradigm for the molecular mode of action in which CdtB interferes with PI-3K signaling; this interference has been demonstrated for AaCdt toxicity in host cells including lymphocytes, macrophages, and mast cells (Shenker et al., 2007; Shenker et al., 2010; Shenker et al., 2014; Shenker et al., 2016). Central to this mechanism of action is AaCdtB’s ability to function as a PIP3 phosphatase similar to the degradative enzymatic activities of the 3-phosphatase PTEN and the 5-phosphatase SHIP (Shenker et al., 2007).

PIP3 is a critical signaling lipid that is utilized by the PI-3K signaling pathway; this pathway is critical to regulation of cell growth, proliferation, and survival, as well as a number of other functions that are specific to individual cell types (reviewed in (Krystal, 2000; Mitsiades et al., 2004; Buckler et al., 2008; Huang and Sauer, 2010; Manning and Toker, 2017)). In general, activation of PI-3K leads to increased production of PIP3 and downstream activation of the pathway; similarly, depletion of PIP3 results in blockade of the pathway, leading to altered cell function(s) that is typically opposite to the effect of pathway activation. A critical event resulting from PIP3 depletion and representative of PI-3K blockade is reduced phosphorylation of the kinases Akt and GSK3β; this results in Akt inactivation and GSK3β activation. To date, we have demonstrated that AaCdtB is a potent PIP3 phosphatase, and, moreover, that AaCdt-treated lymphocytes exhibit significant reductions in PIP3 as well as pAkt and pGSK3β (Shenker et al., 2007; Shenker et al., 2016). It should also be noted that PI-3K blockade, in general, and decreases in the phosphorylation status of these kinases, in particular, are consistent with Cdt toxicity observed in human lymphocytes: decreased cell proliferation and cell survival. Furthermore, these observations are mechanistically significant as Cdt holotoxin comprised of CdtB mutant proteins deficient in phosphatase activity also lack the ability to induce PI-3K blockade and toxicity: cell cycle arrest and apoptosis (Shenker et al., 2016).

As noted earlier, the primary goal of this study was to determine if the toxicity of other Cdts was dependent upon the same critical events as those identified for AaCdt. This includes assessment of CdtB subunits for PIP3 phosphatase activity and *in situ* evidence that this enzymatic activity correlates with concomitant changes associated with blockade of the PI-3K signaling pathway in human lymphocytes. Specifically, we assessed HdCdt, which has >95% sequence homology with AaCdt along with CjCdt for their ability to induce PI-3K blockade; the latter toxin exhibits less sequence homology but similar structural motifs (Eshraghi et al., 2010; Scuron et al., 2016). Indeed, we demonstrate that the CdtB subunits from both HdCdt and CjCdt exhibit potent PIP3 phosphatase activity; this activity was approximately 60% of that exhibited by AaCdtB. Furthermore, Jurkat cells treated with each of these toxins exhibit reductions in both pAkt and pGSK3β. These changes are consistent with a molecular mechanism of action involving PIP3 phosphatase activity associated with the induction of PI-3K signaling blockade.

It should be noted that a critical observation that directly relates to PI-3K signaling blockade is, as we previously demonstrated, that AaCdtB-mediated reductions in pGSK3β translate into measurable increases in enzymatic activity of this kinase (Shenker et al., 2016). Furthermore, Cdt-induced cell cycle arrest was found to be dependent upon GSK3β activation as inhibitors of this kinase blocked G2/M cell arrest. In our current study, we employed two GSK3β inhibitors, LY2090314 and CHIR99021, to determine if GSK3β activity was also necessary for HdCdt and CjCdt. Our findings indicate that HdCdt and CjCdt were also dependent upon GSK3β activity as both inhibitors blocked the ability of these toxins to induce cell cycle arrest, just as previously demonstrated for AaCdt (Shenker et al., 2016).

Previously, we established a critical link between Cdt-mediated PI-3K signaling blockade, the cell cycle regulatory protein p21^CIP1/WAF1^, and toxin-induced apoptosis (Shenker et al., 2020). Several investigators have demonstrated that AaCdt, HdCdt, *Helicobacter hepaticus* Cdt and *E. coli* Cdt induce increases in p21^CIP1/WAF1^ (Cortes-Bratti et al., 2001b; Sato et al., 2002; Yamamoto et al., 2004; Graillot et al., 2016; Pere-Vedrenne et al., 2017). We advanced these observations by demonstrating that p21^CIP1/WAF1^ played a requisite role in Cdt-induced apoptosis; CdtB-associated lipid phosphatase is required to induce the p21^CIP1/WAF1^ increases that are in turn required to induce pro-apoptotic proteins (Shenker et al., 2020). Moreover, cells deficient in p21^CIP1/WAF1^ expression (Jurkat^p21CIP1/WAF1-^) failed to express these pro-apoptotic proteins (Bid, Bax and Bak) and concomitantly did not become apoptotic. We have now extended these findings to include HdCdt and CjCdt as we demonstrate that Jurkat^p21CIP1/WAF1-^ cells also failed to become apoptotic when exposed to these Cdts.

**Figure 7:**
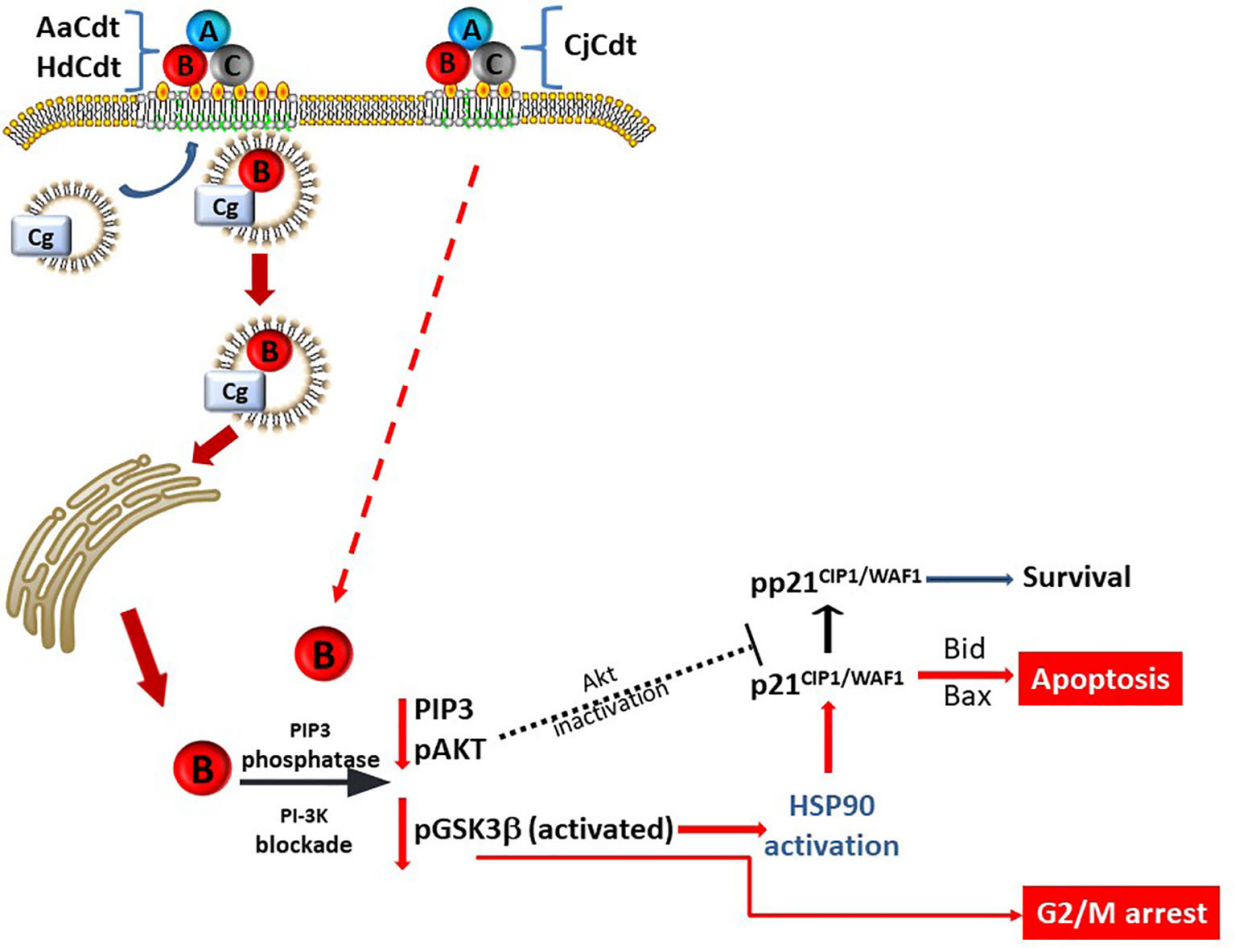
Schematic model showing proposed mechanism for CdtB internalization and toxicity in human lymphocytes. AaCdt, HdCdt, and CjCdt bind to cells *via* cholesterol in the context of membrane lipid rafts. As a result of exposure to Cdt, cellugyrin-containing SLMVs translocate from cytosol to membrane lipid rafts. We propose that this translocation leads to the association of AaCdtB and HdCdtB with the cellugyrin-containing SLMVs. This interaction may involve direct binding to cellugyrin either on extra- or intra-vesicular loops or indirect association *via* an un-identified binding partner. We further propose that CdtB is transported *via* SLMVs to intracellular target sites, such as locations containing PIP3 pools where the enzymatically active CdtB subunit is released from SLMVs and is then able to dephosphorylate the signaling lipid, resulting in PI-3K blockade and toxicity. Based upon CjCdt toxicity independence of cellugyrin for toxicity (and presumably internalization and trafficking), we propose that CjCdtB enters through a cellugyrin- independent mechanism, hijacks retrograde transport mechanisms, and, in lymphocytes, accumulates in sites of PIP3 pools similar to AaCdtB and HdCdtB.

Also noteworthy is that Cdt-induced cell cycle arrest was not dependent upon p21^CIP1/WAF1^ for all three Cdts. Jurkat^p21CIP1/WAF1-^ cells retained the capacity to undergo cell cycle arrest at levels comparable with Jurkat^WT^ cells when treated with each of the Cdts. In extended studies with AaCdt, we reported that the cells remained in G2/M arrest for 48 and 72 hrs [further times were not assessed; (Shenker et al., 2020)]. The dichotomous role of p21^CIP1/WAF1^ in toxin-induced apoptosis versus cell cycle arrest is consistent with our increasing knowledge of the diverse role(s) that this regulatory protein plays in cell function. This includes a role that at times may be pro-apoptotic and at other times anti-apoptotic; the survival function of p21^CIP1/WAF1^ has been reported to be dependent, in part, upon sub-cellular location and phosphorylation status (Kang et al., 1999; Lincet et al., 2000; Gartel, 2005; Ghanem and Steinman, 2005; Abbas and Dutta, 2009; Giordano et al., 2017). Other investigators have demonstrated that p21^CIP1/WAF1^ is a downstream target of pAkt (Zhou et al., 2001; Li et al., 2002b; Zhou and Hung, 2002). In the absence of pAkt, as occurs under CdtB-mediated PI-3K signaling blockade, p21^CIP1/WAF1^ would not be phosphorylated, thereby placing it into a pro-apoptotic mode. Indeed, this is what we observed in previous studies (Shenker et al., 2020). Collectively, these observations suggest that the induction of cell cycle arrest and apoptosis may not be the consequence of sequential, interdependent events; instead, they may be differentially regulated by altered phosphorylation (activity) of downstream kinases of the PI-3K signaling pathway (see **Figure 7**).

Intracellular levels of p21^CIP1/WAF1^ are typically low under normal growth conditions while stress and/or DNA damage are known to induce significant increases. Elevated levels of p21^CIP1/WAF1^ lead to activation of the DDR and G2 checkpoint; these events contribute to cell cycle arrest and initially support survival as cells attempt to repair DNA damage. Indeed, several investigators have reported that Cdt-treated cells exhibit DDR activation (i.e, phosphorylation of H2AX), and further, they have interpreted these findings as evidence of toxin-induced DNA damage (Bezine et al., 2014; Frisan, 2016). However, we have repeatedly demonstrated that the DDR is not activated (ie., we do not observe increases in pH2AX) in lymphocytes under conditions of AaCdt-treatment (Shenker et al., 2006; Shenker et al., 2016; Shenker et al., 2020). In these studies, we employed the minimum doses of toxin required to achieve maximum cell cycle arrest and apoptosis. Under higher doses of toxin, we have observed increases in pH2AX; however, these increases appear to be the result of activation of the apoptotic cascade rather than a direct effect of Cdt on DNA damage. For instance, over expression of the anti-apoptotic protein Bcl-2, blocks Cdt-induced apoptosis and increases in pH2AX (Shenker et al., 2006). We now demonstrate that under similar conditions, neither HdCdt or CjCdt induce increases in lymphocyte levels of pH2AX. These observations are consistent with AaCdt toxicity and suggest that DDR, and hence, DNA damage, are not a component of toxin induced cell cycle arrest and apoptosis in lymphocytes. This raises the possibility that Cdts may utilize different mechanisms in other cells, perhaps due to the requirement for higher doses of toxin to achieve toxicity. This may explain, for example, that Cdt toxicity of some non-lymphoid cells such as epithelial cells may involve DDR activation (Li et al., 2002a; Gargi et al., 2013; Ge et al., 2017; Pere-Vedrenne et al., 2017). Clearly, this is a topic requiring further investigation.

Previously, we demonstrated that p21^CIP1/WAF1^ increases were not associated with a rise in mRNA levels and our observations also exclude a role for DNA damage. Thus, our recent observations regarding the role of heat shock protein HSP90 in AaCdt toxicity are particularly relevant (Shenker et al., 2020). Mechanistically, HSP90 has been shown to stabilize p21^CIP1/WAF1^ and thereby prevent its degradation (Jascur et al., 2005; Diehl et al., 2009). We now demonstrate another similarity between AaCdt and both HdCdt and CjCdt as pretreatment of Jurkat cells with GA, a HSP90 inhibitor, blocked Cdt-induced apoptosis by both HdCdt and CjCdt. It should also be noted that in previous studies we determined AaCdtB-mediated increases in HSP90 were dependent upon retention of its lipid phosphatase activity.

In a final comparison of Cdts, the requirement for the host cell protein, cellugyrin, was assessed. We have demonstrated previously that shortly after holotoxin binding to human lymphocytes and macrophages, CdtB is internalized *via* its association with cholesterol rich membrane microdomains (Boesze-Battaglia et al., 2006; Boesze-Battaglia et al., 2009; Boesze-Battaglia et al., 2015; Boesze-Battaglia et al., 2016). Internalization leads to an association of CdtB with cellugyrin, a component of synaptic-like microvesicles (SLMVs) which may participate in intracellular transport (Boesze-Battaglia et al., 2017; Boesze-Battaglia et al., 2020). Moreover, reduced expression of cellugyrin protects cells from CdtB internalization and subsequent toxicity. Interestingly, in this study we demonstrate that HdCdt shares a similar dependence on cellugyrin to that observed with AaCdtB. In contrast, CjCdt did not exhibit a dependence on this host cell protein as Jurkat^Cg-^ cells exhibit comparable susceptibility to toxicity, including both cell cycle arrest and apoptosis. These findings suggest that AaCdt and HdCdt likely share similar modes of internalization, and, possibly, retrograde transport while CjCdt may utilize different pathways of entering cells and/or hijacking of transport mechanisms (**Figure 7**). This interpretation is consistent with the findings of Gargi et al. (Gargi et al., 2012; Gargi et al., 2013), who demonstrated that HdCdt and EcCdt are transported within cells by distinct pathways.

Our findings that CdtB from multiple Cdts is capable of exhibiting lipid phosphatase activity are not surprising as this subunit is expressed universally within the family of Cdts. Moreover the “family” of CdtBs share similar amino acid sequence homology and/or structural motifs, with respect to catalytic domains (Dlakic, 2000; Dlakic, 2001). This study expands our initial findings of a novel paradigm first reported for AaCdt for CdtB’s molecular mode of action (summarized in **Figure 7**). The ability to exhibit PIP3 phosphatase activity and induce PI-3K signaling blockade is clearly shared by HdCdt and CjCdt. We propose that the toxic effect of these Cdts, at least with respect to human lymphocytes, involves PI-3K signaling blockade and dependence on GSK3β activation. Additionally, blockade of this signaling pathway is also responsible for increases in p21^CIP1/WAF1^ levels and all three of the Cdts are dependent upon these increases to induce apoptosis, but not cell cycle arrest. Clearly, our results do not eliminate a potential role for of CdtB- associated DNase activity in mediating toxicity in some host target cells.

In conclusion, we propose that Cdts represent a class of virulence factors that contribute to the pathogenesis of mucocutaneous infections caused by a wide range of pathogens. The ability of one family of toxins, at least AaCdt, HdCdt and CjCdt, to contribute to a diverse range of disease pathogenesis is consistent with two molecular aspects of Cdt’s mode of action. First, several Cdts utilize a ubiquitous receptor, cholesterol, and are thereby able to bind, internalize and intoxicate a wide range of cell types. Second, the PIP3 phosphatase activity exhibited by CdtB enables the toxin to interfere with a universal signaling pathway that is utilized by virtually all cells. The regulatory role that this pathway plays with respect to individual cell function(s) dictates the adverse (and varied) outcome of toxin-host cell interaction (reviewed in Scuron et al., 2016). Thus, it is not surprising that Cdts have been shown to promote infection by altering epithelial barrier protection, promote inflammatory responses and impair acquired immunity and thereby promote immune evasion. It should be noted that all strains of bacteria do not express Cdt, yet in some instances appear to share similar pathogenicity. Clearly, this discrepancy needs further investigation as it may simply reflect varying roles for Cdt under different conditions. Alternatively, it is possible that studies conducted in animal models often do not recapitulate all facets of disease; thus, it is possible that the virulence of Cdt is less significant under these conditions. Nonetheless, it is intriguing to propose that advancing our understanding of the molecular basis of Cdt toxicity will provide the underpinnings for developing novel approaches for pharmacologic intervention in treating infections associated with Cdt-producing organisms.

## Data Availability Statement

The original contributions presented in the study are included in the article/supplementary material. Further inquiries can be directed to the corresponding author.

## Author Contributions

GH, KB-B, and BS conceptualized the study. KB-B and BS provided oversight and funding acquisition for the study. GH, LW, AZ, and ZS contributed to the methodology. GH and LW contributed to data and figure curation. GH and BS wrote the original draft. KB-B and SB contributed to the writing, review and editing of the manuscript. All authors contributed to the article and approved the submitted version.

## Funding

This work has been supported by grants DE006014 and DE023071 from the National Institute of Dental and Craniofacial Research at the National Institutes of Health.

## Conflict of Interest

The authors declare that the research was conducted in the absence of any commercial or financial relationships that could be construed as a potential conflict of interest.
